# Use of Inactivated Polio Vaccine Among U.S. Adults: Updated Recommendations of the Advisory Committee on Immunization Practices — United States, 2023

**DOI:** 10.15585/mmwr.mm7249a3

**Published:** 2023-12-08

**Authors:** Sarah Kidd, Thomas Clark, Janell Routh, Sybil Cineas, Lynn Bahta, Oliver Brooks

**Affiliations:** ^1^Division of Viral Diseases, National Center for Immunizations and Respiratory Diseases, CDC; ^2^Warren Alpert Medical School of Brown University, Brown University, Providence, Rhode Island; ^3^Minnesota Department of Health; ^4^Watts Healthcare Corporation, Los Angeles, California.

**Summary**

**What is already known about this topic?**

Previously, inactivated polio vaccine (IPV) recommendations for U.S. adults addressed adults known to be at increased risk for poliovirus exposure.

**What is added by this report?**

On June 21, 2023, the Advisory Committee on Immunization Practices issued an IPV recommendation for all adults known or suspected to be unvaccinated or incompletely vaccinated against polio. Risk-based recommendations for IPV boosters have not changed.

**What are the implications for public health practice?**

Adults aged ≥18 years who are known or suspected to be unvaccinated or incompletely vaccinated against polio should complete a primary polio vaccination series with IPV. Fully vaccinated adults at increased risk for poliovirus exposure may receive a single lifetime booster dose of IPV.

## Abstract

Poliovirus can cause poliomyelitis and lifelong paralysis. Although wild poliovirus types 2 and 3 have been eradicated, wild poliovirus type 1 and vaccine-derived polioviruses are still circulating in multiple countries worldwide. In 2022, a case of paralytic polio caused by vaccine-derived poliovirus type 2 was identified in an unvaccinated young adult in New York. This case and subsequent detection of community transmission underscored the ongoing risk for importation of poliovirus into the United States and risk for poliomyelitis among unvaccinated persons. However, previous Advisory Committee on Immunization Practices (ACIP) recommendations for adult polio vaccination were limited to adults known to be at increased risk for exposure. During October 2022–June 2023, the ACIP Polio Vaccine Work Group reviewed data on poliovirus surveillance and epidemiology, safety and effectiveness of inactivated poliovirus vaccine (IPV), and other considerations outlined in the ACIP Evidence to Recommendations Framework. On June 21, 2023, ACIP voted to recommend that all U.S. adults aged ≥18 years who are known or suspected to be unvaccinated or incompletely vaccinated against polio complete a primary polio vaccination series with IPV. This report summarizes evidence considered for this recommendation and provides clinical guidance for the use of IPV in adults.

## Introduction

Poliovirus infection can cause poliomyelitis and permanent paralysis. The incidence of paralytic polio in the United States decreased rapidly after introduction of the Salk inactivated poliovirus vaccine (IPV) in 1955 followed by the Sabin oral poliovirus vaccine (OPV) in 1961 (*1*). Trivalent OPV (tOPV), containing poliovirus vaccine serotypes 1, 2, and 3, was administered as part of the routine childhood immunization schedule starting in the 1960s and led to the elimination of wild poliovirus and community poliovirus transmission in the United States in 1979. In 1996, the current enhanced-potency formulation of IPV was introduced as part of a sequential vaccination schedule with tOPV. In 1999, the United States adopted an IPV-only schedule, removing tOPV. Since then, IPV has been the only polio vaccine recommended for routine immunization in the United States.

Historically, the Advisory Committee on Immunization Practices (ACIP) has not recommended polio vaccination for persons aged ≥18 years unless they are known to be at increased risk for poliovirus exposure* (*2*). In 2022, a case of paralytic polio caused by circulating vaccine-derived poliovirus type 2 (cVDPV2) was identified in an unvaccinated young adult in New York (*3*,*4*). Shortly thereafter, retrospective and prospective wastewater testing detected poliovirus type 2 genetically linked to the case in six New York counties during April–October 2022 (*5*), indicating community circulation. Genetic sequencing subsequently demonstrated linkages between the New York virus and polioviruses collected from wastewater in Canada, Israel, and the United Kingdom. Rockland County, New York has reported low rates of childhood vaccination for >20 years; in the summer of 2022, 60% of Rockland County children aged <2 years had received the recommended 3 doses of IPV, and coverage in some county zip codes was as low as 37%. In comparison, national 3-dose IPV coverage by age 2 years was 93.4% among children born during 2018–2019 (*6*).

These events represent only the second known instance of community transmission of poliovirus in the United States since 1979. The occurrence of this paralytic polio case, along with ongoing global poliovirus circulation and risk for future poliovirus importations into the United States, prompted a reexamination of polio vaccination recommendations and guidance for U.S. adults, particularly those who are known to be unvaccinated or incompletely vaccinated.

## Methods

The ACIP Polio Vaccination Work Group includes clinicians and experts in infectious diseases, vaccinology, and public health. During October 2022–June 2023, the Work Group met at least monthly to discuss adult IPV recommendations using the ACIP Evidence to Recommendations Framework^†^ to guide deliberations. The framework considerations included polio as a public health problem, resource use, benefits and harms of vaccination, patient values and preferences, acceptability, feasibility, and equity. Deliberations included review of poliovirus surveillance and epidemiologic information, as well as published data on IPV safety and effectiveness identified through literature searches. A summary of the Work Group’s deliberations and conclusions was presented to ACIP at a public meeting on June 21, 2023.

## Rationale and Evidence: Unvaccinated and Incompletely Vaccinated Adults

The immunogenicity and effectiveness of enhanced-potency IPV has been established; the presence of neutralizing antibodies correlates with protection against paralytic disease (*7*). Seroconversion rates and antibody titers after vaccination vary depending on age at receipt of the first dose and vaccination schedule, but administration of 3 IPV doses ≥2 months apart to children aged ≥2 months results in ≥95% seroconversion 1 month after receipt of the third dose (*8*,*9*). In contrast to OPV, IPV does not prevent gastrointestinal infection or shedding in exposed persons (*10*); however, IPV does appear to reduce the odds of nasopharyngeal shedding in infected persons (*11*,*12*).

During >20 years of use in routine immunization, the current formulation of enhanced-potency IPV has been demonstrated to have a highly favorable safety profile. Local reactions at the injection site are the most commonly reported adverse events, with 14%–29% of clinical trial recipients reporting tenderness at the injection site (*13*). Concurrent administration of IPV with other vaccines was not associated with increased frequency of adverse events or severity of adverse events compared with administering the other vaccines alone (*8*,*14*,*15*), and no serious adverse events have been causally associated with the current IPV formulation (*15*–*17*).

The most recent ACIP statement on adult polio vaccination was published in 2000 and recommended IPV for unvaccinated and incompletely vaccinated adults who were at increased risk for exposure to poliovirus (*2*). However, this recommendation did not directly address other unvaccinated and incompletely vaccinated adults. The detection of a paralytic polio case caused by cVDPV2 in Rockland County, New York in July 2022 (*3*,*4*) demonstrated that adults are living in the United States who are known to be unvaccinated or incompletely vaccinated and that they are frequently clustered together in communities that also have low childhood vaccination rates. The events in New York also served as a reminder of the risk for importation of poliovirus into the United States as long as any polioviruses are circulating globally. A uniform recommendation for all adults who are known or suspected to be unvaccinated or incompletely vaccinated would allow these adults to benefit from opportunities to receive IPV vaccination and be protected from paralytic polio before they are at risk for exposure.

## Rationale and Evidence: Booster Doses for Previously Vaccinated Adults

A national serosurvey conducted during 2009–2010 determined that ≥79% of adults aged 20–49 years have antibodies to poliovirus types 1, 2, and 3 (*18*), indicating the persistence of antibodies for at least several decades. No data on comparative vaccine effectiveness of a primary series alone versus a primary series plus booster IPV dose exist; however, studies in groups of adults with varying vaccination histories and a range of prebooster seroprevalences have demonstrated that administering an IPV booster dose increases the percentage of adults who are seropositive to 98%–100% (*19*–*24*). Although the need for an IPV booster after primary polio vaccination is uncertain, some adults might benefit from the increased immunity provided by an additional IPV dose when exposure to poliovirus can reasonably be expected. Therefore, adults who have completed a primary series of tOPV or IPV and who are at increased risk for exposure to poliovirus may receive another dose of IPV. This recommendation is unchanged from the previous booster recommendation (*2*).

## Recommendations

### Unvaccinated or Incompletely Vaccinated Adults

Adults aged ≥18 years who are known or suspected to be unvaccinated or incompletely vaccinated against polio should complete a primary vaccination series with IPV.

### Vaccinated Adults Who are at Risk for Exposure to Poliovirus

Adults who have received a primary series of tOPV or IPV in any combination and who are at increased risk for exposure to poliovirus may receive another dose of IPV. Available data do not indicate the need for more than a single lifetime booster dose with IPV for adults.

## Clinical Considerations

Polio vaccination has been part of routine childhood immunization since the late 1950s. Adults who received any childhood vaccines almost certainly were vaccinated against polio. Thus, most adults who were born and raised in the United States can assume they were vaccinated against polio as children, even if they do not have written documentation of vaccination, unless they have specific reasons to believe they were not vaccinated. The current definition of a complete primary polio vaccination series is receipt of ≥3 appropriately spaced doses of tOPV or IPV in any combination, with the final dose in the series administered on or after the fourth birthday.^§^

### Persons at Increased Risk for Poliovirus Exposure

Adults who might be at increased risk for exposure to poliovirus include travelers to countries where polio is epidemic or endemic, laboratory and health care workers who handle specimens that might contain polioviruses, health care workers or other caregivers who have close contact with patients in a community with a polio outbreak, and other adults who are identified by public health authorities as being part of a group or population at increased risk for exposure to poliovirus because of an outbreak.

### Dosing Schedule

Adults requiring a primary polio vaccination series should receive 2 doses of IPV administered at an interval of 4–8 weeks; a third dose should be administered 6–12 months after the second dose. There is no need to restart the series if the interval between doses exceeds the recommended interval. If 3 doses of IPV cannot be administered within the recommended interval before protection is needed (e.g., before travel to a country with endemic polio), an accelerated schedule is recommended based on the amount of time available.^¶^

### Considerations for Persons with Altered Immunocompetence

IPV is an inactivated vaccine and is safe to administer to persons who are immunocompromised or who have close contact with other persons who are immunocompromised. However, IPV might be less effective when administered during periods of altered immunocompetence. For this reason, when feasible, IPV should be administered before initiation of immunosuppressive therapy or anticipated period of altered immunocompetence. Specifically, for persons anticipated to be eligible for an IPV booster in the future (e.g., before travel to a country with endemic polio), administration of the booster dose before the period of altered immunocompetence should be considered. Additional guidance regarding immunization in persons with specific conditions is available at https://www.cdc.gov/vaccines/hcp/acip-recs/general-recs/immunocompetence.html.

### Contraindications and Precautions

Contraindications and precautions are unchanged from previous recommendations. Severe allergic reaction (e.g., anaphylaxis) to IPV or to antibiotics contained in trace amounts in IPV (streptomycin, polymyxin B, or neomycin) is the only contraindication to administration of IPV. Pregnancy is a precaution to administration of IPV. Although there is no evidence that IPV vaccine causes harm to pregnant persons or their fetuses, out of an abundance of caution IPV should not be given during pregnancy if there is not an increased risk for exposure. However, if a pregnant person is at increased risk for exposure and requires immediate protection against polio, IPV can be administered in accordance with the recommended schedule for adults (*2*).

### Reporting of Vaccine Adverse Reactions

Adverse events occurring after administration of any vaccine should be reported to the Vaccine Adverse Event Reporting System (VAERS). Reports can be submitted to VAERS online, by fax, or by mail. Additional information about VAERS is available by telephone (1-800-822-7967) or online at vaers.hhs.gov.

## Footnotes

* In the 2000 ACIP statement on polio vaccination, persons who were at higher risk for poliovirus exposure than the general population included travelers to areas or countries where polio is epidemic or endemic, members of communities or specific population groups with disease caused by wild polioviruses, laboratory workers who handle specimens that might contain polioviruses, health care workers who have close contact with patients who might be excreting wild polioviruses, and unvaccinated adults whose children will be receiving oral poliovirus vaccine.

† Evidence to Recommendation documents are available for adult primary vaccination with IPV (https://www.cdc.gov/vaccines/acip/recs/grade/primary-IPV-polio-vax-adults-etr.html) and for the adult IPV booster dose (https://www.cdc.gov/vaccines/acip/recs/grade/booster-IPV-polio-vax-adults-etr.html).

§ The recommendation for a dose on or after the fourth birthday was made in August 2009. Therefore, persons who received ≥4 doses of tOPV or IPV before August 2009 may be considered fully vaccinated, regardless of the age of the final dose.

¶ If >8 weeks are available before protection is needed, 3 doses of IPV should be administered ≥4 weeks apart. If <8 weeks but >4 weeks are available before protection is needed, 2 doses of IPV should be administered ≥4 weeks apart. If <4 weeks are available before protection is needed, a single dose of IPV is recommended. The remaining doses of vaccine should be administered later, at the recommended intervals.
